# Population-based guiding for evolutionary neural architecture search

**DOI:** 10.1038/s41598-025-25840-5

**Published:** 2025-11-07

**Authors:** Stefan Dendorfer, Andreas M. Kist

**Affiliations:** Department Artificial Intelligence in Biomedical Engineering, Friedrich-Alexander-Universität Erlangen-Nürnberg, Erlangen, Germany

**Keywords:** Neural architecture search, Guided evolution, Benchmarks, Computational biology and bioinformatics, Evolution, Mathematics and computing

## Abstract

Neural Architecture Search (NAS)—combined with biology-inspired evolutionary methods—can help discover suitable architectures tailored to a given objective. A guided evolutionary approach can enhance efficiency, aiming to accelerate the discovery of top-performing architectures within a given search space. We propose a novel algorithmic framework that implements selection, crossover, and mutation operations to generate new candidate architectures during an evolutionary Neural Architecture Search: A greedy selection operator, relying solely on model accuracy data, promotes exploitation. Incorporating architecture embeddings to further refine the mutation process enhances exploration. We introduce a guided mutation approach to steer the search toward unexplored regions of the current population. The proposed strategy, PBG (Population-Based Guiding), synergizes both explorative and exploitative methods. It substantially outperforms baseline methods such as regularized evolution by being up to three times faster on NAS-Bench-101. This combined approach not only leverages the strengths of both explorative guided mutation and exploitative greedy selection strategies, but also provides a robust and efficient framework reaching competitive performance for evolutionary Neural Architecture Search across benchmarks.

## Introduction

While AutoML can optimize hyperparameters to improve the performance of any given neural network, one can also improve the neural network itself to enhance performance. Instead of manually designing neural network architectures, Neural Architecture Search (NAS) can automatically discover high-performing neural architectures within a given search space. For example, biology-inspired evolutionary strategies excel by adapting to diverse neural network design requirements^1^. They perform a robust, gradient-free global search that handles multiple objectives and incorporates domain knowledge. This makes them particularly effective for exploring vast, complex search spaces^2^.

However, traditional evolutionary NAS methods remain inefficient. Despite their success, they still rely heavily on handcrafted algorithmic components, such as manually tuned mutation rates or fixed selection strategies. These human-designed heuristics introduce bias and limit adaptability. Moreover, evolutionary methods typically require a large number of candidate evaluations, making them computationally expensive. As search spaces continue to grow exponentially with network depth and connectivity, brute-force or naively guided approaches quickly become infeasible, even with modern hardware.

Recent advances^3^ have shown that minimizing human bias in architecture design allows NAS algorithms to rediscover and surpass expert-designed networks^4^. Yet, the bias at the meta-level–the design of the NAS algorithm itself–remains largely unaddressed. Current methods often balance exploration and exploitation through static heuristics rather than adaptive mechanisms, leading either to premature convergence or inefficient search. This highlights a key gap: how to make evolutionary NAS algorithms self-guiding, adaptive, and computationally efficient without introducing new hyperparameters. Modern studies focus on Zero-Shot NAS methods that eliminate the need for full training by leveraging proxy metrics, significantly reducing computational costs^5^. However, these methods often lose the flexibility and generality of evolutionary approaches. A promising direction is therefore to combine evolutionary search with lightweight guidance mechanisms that dynamically exploit population knowledge to steer evolution toward promising regions of the search space–without relying on external supervision or expensive model training.

In this work, we address these challenges by proposing a Population-Based Guiding (PBG) framework that integrates greedy selection, random crossover, and a novel guided mutation mechanism. Our method aims to eliminate human bias in algorithm design, balance exploration and exploitation adaptively, and achieve high efficiency across diverse benchmarks.

This work focuses on the following contributions:

- Promoting exploitation through greedy selection, which selects the best parent pairs based on combined fitness for reproduction.
- Introducing a novel, parameter-free guided mutation algorithm that uses the current population of a generation to propose mutation indices for exploration.
- Achieving competitive and robust performance and generalizability in evolutionary Neural Architecture Search through PBG (Population-Based Guiding), a holistic approach that leverages greedy selection, random crossover, and guided mutation.

## Related work

### Evolutionary neural architecture search

 NAS is a search problem that aims to find a well-performing architecture *A* within a search space $\mathcal {A}$ using a search strategy that selects candidates to be evaluated by a performance estimation strategy^6^. The search strategy can be categorized into black-box optimization and one-shot techniques^7^. One-shot techniques, including differentiable methods such as DARTS^8^, are computationally efficient and have shown promising results. Among black-box methods, evolutionary or genetic algorithms stand out by iteratively generating new architectures through crossover and mutation of selected architectures. In the trade-off between exploration and exploitation, selection can leverage evaluated architectures, and crossover recombines successful individuals’ genetic material, fostering both^9^, while mutation introduces novelty to explore the search space^10–12^. Novel methods in NAS show promising results, such as regularizing evolution by dropping the oldest architecture (concept of aging) in a population^13^. However, there is still potential for improvement by enhancing the genetic algorithm itself. Multiple methods exist for selection^14–17^, crossover^18–21^, and mutation^19,22,23^. However, only a small fraction holistically approach the problem. Recent advances in evolutionary NAS^2^, which continue to push performance baselines, highlight the need for further exploration into the design of genetic algorithms. A recent approach drops the manual design altogether and meta-learns selection and mutation rate operations in the field of neuroevolution^24^. The idea of guiding genetic algorithms^25^, which integrates domain knowledge or heuristic rules to direct the search into promising regions, laid the foundation for a whole domain of guided genetic algorithms^26,27^. Performance-based guiding can leverage fitness by prioritizing the evolution of solutions that perform better, leading to a focus on exploiting high-performing candidates. Building on the idea of performance-based guiding, a closely related paper couples a zero-proxy estimator with the search method, forcing further exploitation of favorable settings^28^. Our proposed greedy selection algorithm differs by using already pre-evaluated parent architectures, eliminating the need for zero-proxy scoring to guide the selection of parent pairings. Search space landscape-based guidance analyzes and models the search space, as seen in Population-Based Incremental Learning (PBIL). While common methods focus on enforcing diversity in the population^29,30^, our guided mutation differs by directing mutation toward unexplored regions in the search space.

### Population-based incremental learning

 Unlike evolutionary strategies, PBIL refines a single probability vector over generations, representing distributions over solution components, which is updated based on the performance of sampled architectures. This approach thus eliminates the need for genetic operators^31^. In NAS, PBIL balances exploration and exploitation more efficiently than genetic algorithms by directly updating the probability distribution^31^. Recent hybrid PBIL approaches integrate gradient and reinforcement learning updates^21^, bridging probabilistic optimization and differentiable NAS methods. Zhang et al.^27^ enhance PBIL by using a probability vector to guide mutations on existing solutions, updating the vector based on the parent set’s center with a learning rate. The proposed approach builds on this by using the current population to guide mutation instead of relying on a historical probability vector, essentially reshuffling the vector to eliminate the need for a learning parameter.

## Methods

**Fig. 1:**
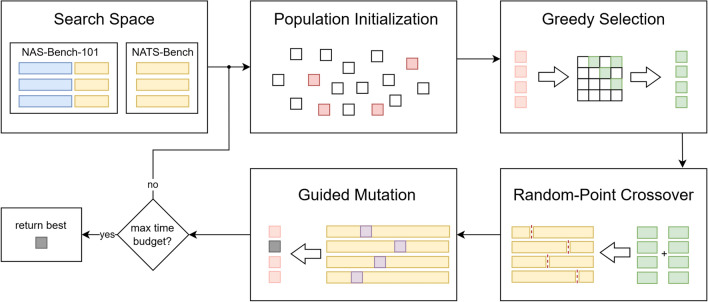
Schematic overview. For the respective search space, a population of individuals gets initialized, selected, crossed, and mutated until the maximum time budget is reached.

In the following, we describe PBG (Population-Based Guiding), a holistic algorithm that combines greedy selection, a fixed-point crossover by randomly sampling a crossover point, and guided mutation. A schematic is shown in Fig. 1.

Greedy selection prioritizes exploitation, while the random crossover point facilitates a balance between randomness and reuse of established components. Finally, guided mutation steers the population toward exploring new territories. This combined approach effectively addresses the trade-off between exploration and exploitation, harnessing synergies from both strategies.

### Greedy selection

Our selection process consists of two phases: candidate selection and pairing selection. Unlike typical greedy selection methods, which focus only on selecting the best individuals, we combine these phases into a unified approach that simultaneously selects candidates and pairs them.

**Fig. 2:**
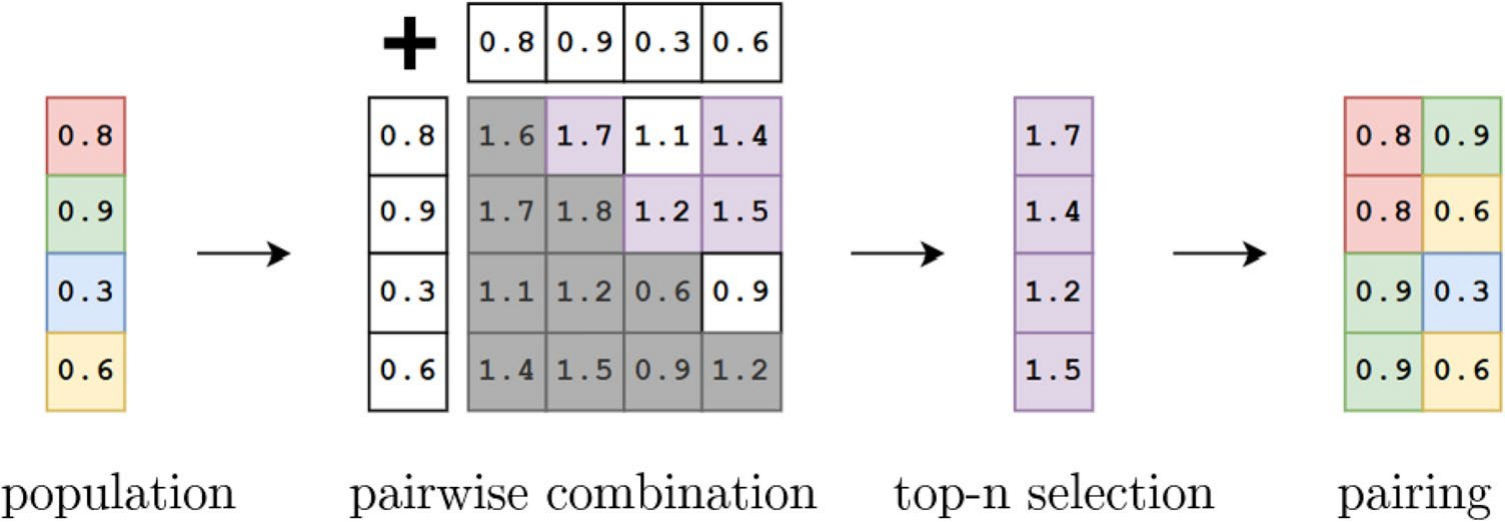
Schematic of greedy selection. A given population with performance metrics (e.g. accuracy) is combined in a pairwise fashion and top-n candidates are selected and used for pairing in the next generation.

Given a population of *n* individuals, we generate all possible pairings, excluding self-pairing and permutations, resulting in ${n(n - 1)}/{2}$ combinations. Each pair is assigned a score by summing the fitness of the two individuals. Fitness here can be any floating-point metric, such as training accuracy, representing a score for the entire individual model. The top *n* pairings with the highest combined fitness scores are selected for reproduction. A schematic is shown in Fig. 2.

This method differs from traditional greedy selection, where only the fittest individuals are selected and paired randomly or sequentially. By pairing candidates based on the best combinations rather than fitness alone, we increase diversity and reduce the risk of premature convergence. The result is a more explorative selection process that maintains strong candidates while fostering greater variation in the population. Unlike conventional techniques that use probabilistic selection based on fitness or rank, this method is purely greedy and exploitative, focusing entirely on maximizing performance without relying on randomness or probabilities.

### Guided mutation

This approach guides mutation locations based on the current population, eliminating the need for target selection (i.e., choosing specific regions to guide mutation toward) and the need for hyperparameter tuning of mutation rates. Using the current population to guide mutations reduces randomness and fosters more informed decisions.

**Algorithm 1:**
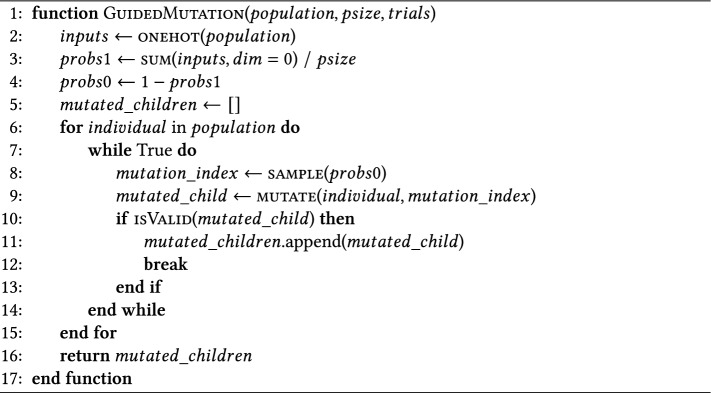
Population-based guided mutation.

**Fig. 3:**
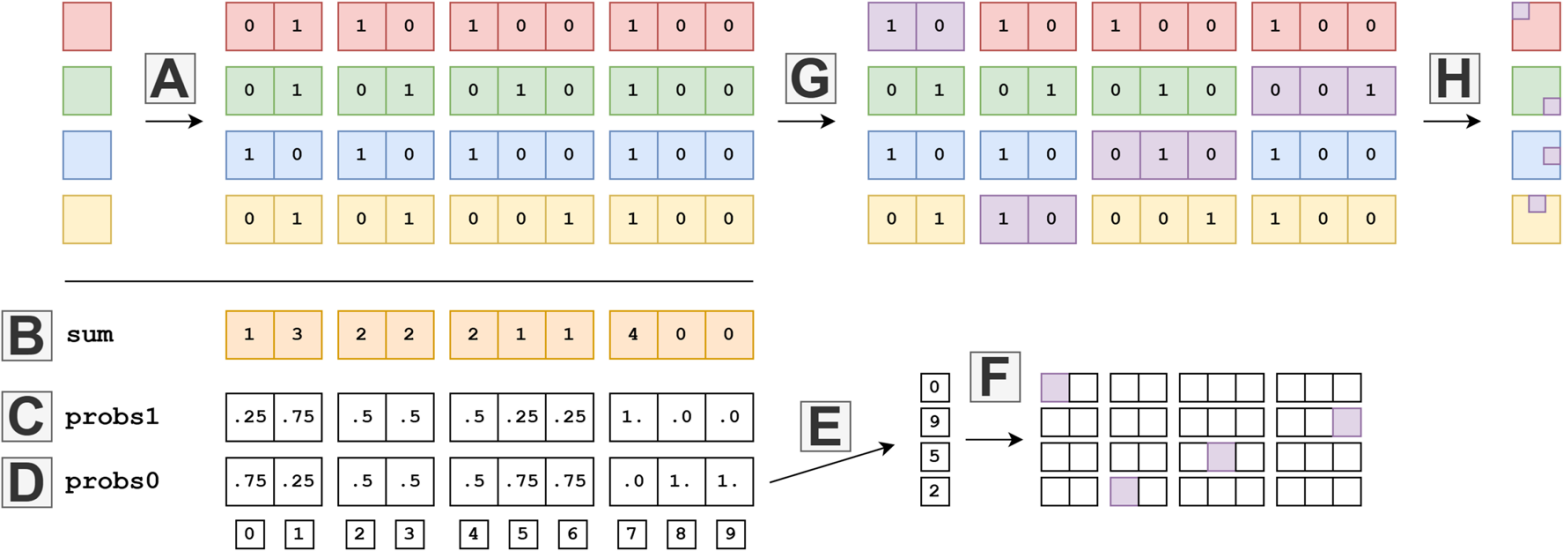
Visualization of calculating the probability vectors from a population of 4 individuals with one-hot encoding. (**A**) Extracts one-hot encoded individuals. (**B**) Sums the population over n individuals. (**C**) Divides sum over number of individuals. (**D**) Calculates its inverse by subtracting from one. (**E**) Samples n times from this probability vector. (**F**) Encodes the sample as positions. (**G**) Applies positions to mutate categorical encoding. (**H**) Returns mutated individuals.

Algorithm 1 describes the population-based guided mutation, which uses a categorical one-hot encoding, now represented as binary vectors, for each individual of the population. A schematic visualization of calculating the mutation indices from four individuals in a population is provided in Fig. 3. The inputs are created from a flattened, categorical one-hot representation of each individual encoded architecture in the population (A). This categorical encoding could represent binary categories for connections (e.g., first column group) or multicategorical for operations (e.g., third column group). These values are summed (B) and averaged along the population count axis to form the vector probs1 (C). The inverse, probs0 (D), is then computed by subtracting probs1 from a vector of ones (i.e., probs0 = 1 − probs1).

Sampling the mutation indices (E) from the probs1 vector (PBG-1 variant) would be functionally similar to the approach proposed in ^27^. This can be interpreted as an exploitation of the current population and applying the Proximate Optimality Principle (POP)^32^, assuming that similar solutions have similar fitness.

This work proposes the novel approach of sampling from the inverted vector, i.e., probs0 (PBG-0 variant), to encourage the exploration of less-visited regions of the population. For every individual member of the population, an index is sampled based on the distribution of the population (F). This algorithm can easily be extended to introduce a mutation rate of sampling more than one index at a time for a more elaborate sampling scheme like in ^27^. Indices with high occurrences of zeros are thus more likely to be sampled than high occurrences of ones. In the example in Fig. 3, the second operation (fourth column group) of the probs0 vector ([0., 1., 1.] indicates high probabilities for sampling the second or third operation type as mutation index, which are not present in the current population. The indices represent the locations in the one-hot encoded genotype. While maintaining the categorical encoding by first setting the surrounding categories of the index to 0, the mutation index is then set to 1 via the mutate function (G). An inner while loop is designed to check for valid mutated specifications, which is exited (H) when a valid mutation for an individual is found. Leveraging both probs1 and probs0 can guide mutation locations based on the current population, eliminating the need for target selection and hyperparameter tuning of mutation rates. This method is yet to be fully studied, with further details available in the ablation studies in Section Exploitative guided mutation on operations drives performance gains.

## Experiments

### Datasets

Tabular NAS benchmarks, such as NAS-Bench-101^33^ and NATS-Bench^34^, provide a standardized framework that enhances reproducibility, accessibility, and comparability across different NAS methods^33^. The primary advantage of using tabular benchmarks^35^ is the efficient use of precomputed data, which significantly reduces the time required for architecture evaluation. This allows researchers to focus on algorithm development and makes it easier to perform statistically significant comparisons of average run performances. Additionally, these benchmarks ensure robust testing, promoting reproducibility and reliability^36^. In all experiments, 1000 runs were averaged in accordance with the guidelines proposed in ^33,36,37^. NAS-Bench-101 consists of 423,624 neural networks trained on CIFAR-10 for 108 epochs, using 1$\times$1 and 3$\times$3 convolutions, and 3$\times$3 max pooling in a cell-based search space^33^. The topology search of NATS-Bench includes 15,625 possible architectures using 5 operations: zeroize, skip connection, 1$\times$1 and 3$\times$3 convolutions, and 3$\times$3 average pooling^34^. It evaluates networks on CIFAR-10, CIFAR-100, and ImageNet16-120, providing a controlled comparison setting for NAS methods.

### Encoding

**Fig. 4:**
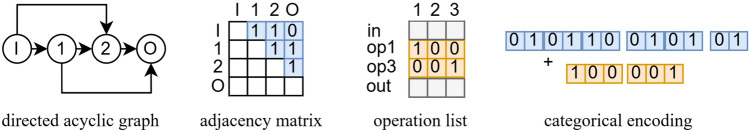
Visualization of encoding on NAS-Bench-101.

For NAS-Bench-101, architectures were encoded using a flattened adjacency matrix and one-hot encoded operations. To ensure consistency, we used categorical encoding, representing each adjacency entry with two bits and operations with a one-hot vector. A visualization is shown in Fig. 4.

**Fig. 5:**
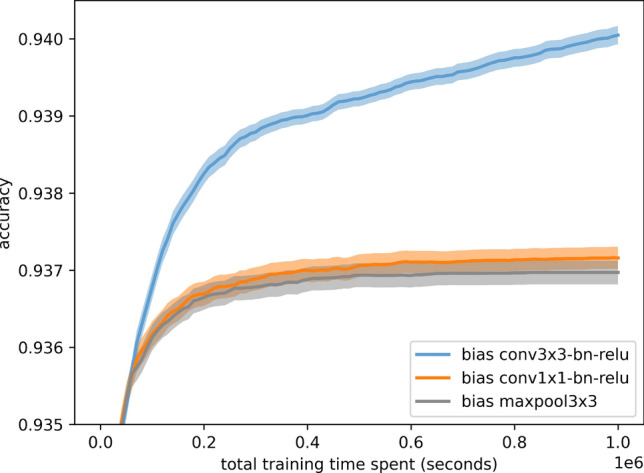
Performance bias when favoring certain operations in NAS-Bench-101 with 95% confidence intervals.

However, closer investigation of the available operations revealed an operation bias: when mutation is biased to favor a specific operation, it leads to different performance outcomes. As shown in Fig. 5, favoring 3x3 convolutions in mutations improves algorithmic performance but poses a risk of overfitting on the benchmark.

For NATS-Bench (topology search space), the encoding differs: each of the 6 possible edges in the graph can represent one of 5 possible operations. This results in a $5 \cdot 6 = 30$-dimensional representation. To support seamless switching between different benchmarks and their specific encodings, we explored a unified encoding approach, which we describe in Section Benchmark unification.

### Shadowed nodes

Node shadowing is a phenomenon that occurs in the context of encoding neural network architectures using a binary adjacency matrix for connections and associating operations with nodes rather than edges. Node shadowing happens when a node is neither directly nor indirectly connected to neither the input nor the output of the network. As a result, the operation assigned to that node becomes irrelevant because the node does not affect the overall computation. When a node is shadowed, its connecting operation is thus also shadowed. Although these nodes are technically pruned and do not directly impact the model’s performance, it is still possible to mutate the shadowed operations. While temporarily shadowed, their nodes may become active in the future if subsequent mutations and crossovers reconnect them, thereby influencing the model’s behavior. This highlights the necessity of implementing a pruning mechanism to identify shadowed nodes and further investigate their effects. A distinction must be made between incoming and outgoing shadowing, leading to a duality in the problem.

**Fig. 6:**
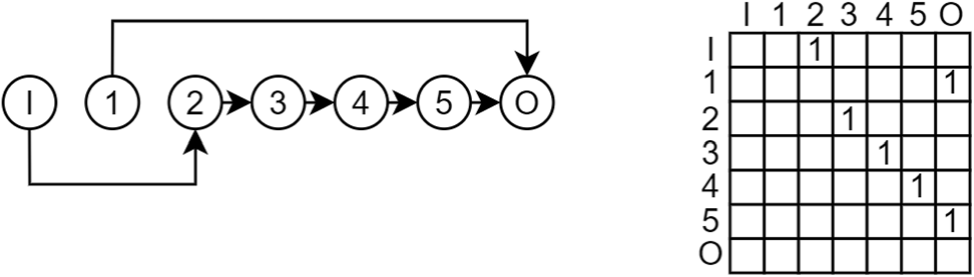
Node 1 is shadowed via incoming shadowing because no other node connects to it as a target. The respective column 1 is all zeros.

**Fig. 7:**
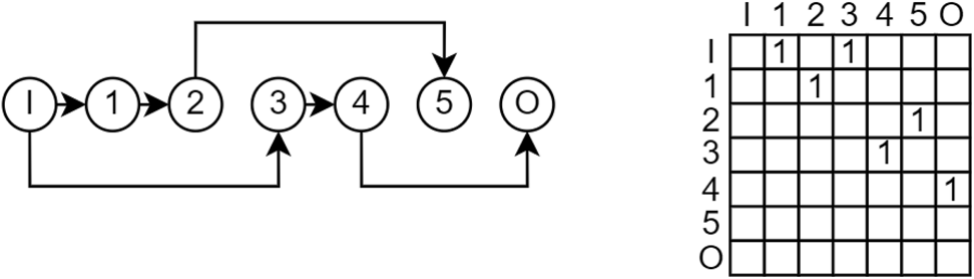
Node 5 is shadowed via outgoing shadowing because it does not connect to any other node as the source. The respective row 5 is all zeros.

Incoming shadowing refers to no preceding nodes being connected to the considered node (direct), as shown in Fig. 6. Conversely, outgoing shadowing refers to neither succeeding nodes nor connection to the output node as shown in Fig. 7.

**Fig. 8:**
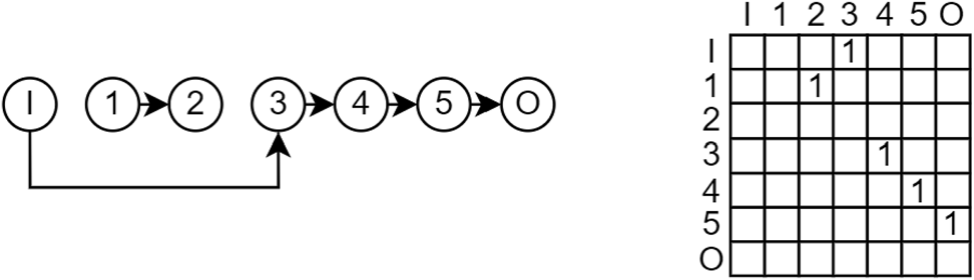
Node 2 is indirectly shadowed because no other node connects to the connected node 1 (incoming shadowing because column 1 is all zeros). Also, node 1 is indirectly shadowed because no other node connects to the connected node 2 (outgoing shadowing because row 2 is all zeros).

The upper triangle adjacency matrix encoding for the directed graph maps node connections from the origin node (row) to the destination node (column). Detecting incoming shadowing is as simple as checking if the column of the considered node consists entirely of zeros, indicating that no other node connects to it. Similarly, outgoing shadowing is identified by checking if the row contains zeros, suggesting no outgoing connections. It is important to note that checking for all zeros in one column or row is sufficient to prove shadowing, but not necessary, as nodes can indirectly be shadowed when connecting to other shadowed nodes themselves. This refers to indirect shadowing, as shown in Fig. 8.

Due to the nature of the DAG, this issue can be resolved iteratively. Starting from the input (output) node for incoming (outgoing) shadowing, the algorithm traverses up (down) the node order, checking the columns (rows) for zeros and ignoring non-zero entries when these nodes have been identified as shadowed before.

For an empirical evaluation of occurrences, we counted the number of shadowing nodes averaged over 1000 runs, yielding 431 mutation processes, with the first node being shadowed 206 times, and in 14 instances, it was the only operation mutated. On average, 2 out of the 5 nodes were shadowed per mutation. Opting not to mutate shadowed nodes, thereby avoiding shadowed operations, can potentially yield advantageous outcomes that could be exploited.

### Benchmark unification

**Fig. 9:**
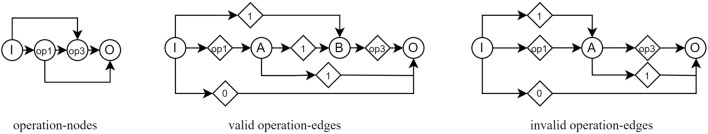
Unification approach of representing the same graph in operation-nodes and operation-edges representation.

A simplified example tries to showcase the unification of the two representations. The operation-nodes representation with adjacency matrix and operation list, as in the NAS-Bench-101 uses nodes as operations and edges as connections. The operation-edges representation uses the operations, including skip connections and zeroizations as edges as in the NATS-Bench. The simplification for operation-nodes representation uses 2 nodes that can represent one of three possible operations, as shown in the left panel of Fig. 9. This brings the adjacency matrix combinations to possibly $2^6$ (6 edges can take values of either 0 or 1) and the operations to $3^2$ (2 nodes can either be op1, op2, or op3) resulting in $2^6 \cdot 3^2$ combinations, not counting graph isomorphisms. Figure 9 shows a unification approach of both representations. To map the operation-nodes representation (left) to an operation-edges representation (middle), two nodes are also required, as one node would lead to two operations for the same edge (right), which is invalid for this representation.

The valid operation-edges representation with two nodes contains 6 possible edges with 5 possible operations (3 original operations plus skip connection and zeroization), resulting in $6^5$ possible combinations. This covers a significantly larger space (7776 vs 576), which results in combinations that are not present in the original operation-nodes representation. This results in a representation mapping that is not a surjective function (injectivity would require careful consideration of graph isomorphisms). A true unification of both representations would require a bijective mapping between the two representations. Applying the operation-edges representation for both datasets would create new architecture configurations that are not included in the original NAS-Bench-101 dataset and can not be evaluated. The application of an operation-nodes representation would reduce the number of architectures from the NATS-Bench dataset significantly, as many architectures can not be transferred to this representation.

### Guiding mutation based on a priori known best models

We investigate how guiding mutation by sampling indices from different groups of architectures - grouped by their performance - affects the search process. Using a pre-evaluated architecture benchmark such as NAS-Bench-101, we can sort all architectures by their performance and then apply different groups of averaged architectures to form a mutation index probability vector. We use averaged groups of the top 1, 10, 100, and 1000 models, selected first from the entire dataset, and then from the dataset after excluding the top 50%. It is important to note that this experiment is intended to investigate the influence of guiding the search when the solutions (i.e., the top models) are known already, making this algorithm ineffective in practice.

### Benchmarking PBG on NAS-Bench-101

Following the guided models that require knowledge of the best models a priori, we iteratively use the most recently evaluated population (which equals the current generation) to guide our mutation. We used greedy selection, random-point crossover, and guided mutation, applying one mutation index for the adjacency matrix and one for operations per individual. We evaluated both variants of guiding mutation based on both probs1 and probs0 for sampling mutation indices. For population size (psize), we chose 50 individuals, as a comparison showed relatively similar results regardless of population size. Table 1 compares algorithms with different population sizes on a time budget of 1*e*7 seconds. Note that these results are based on unoptimized raw data, so the performance indicated in this table may not be fully representative and could be misleading.

**Table 1:** Performance across algorithms on 1000 runs. SEM = 0.00005.

Algorithm	Psize 8	Psize 50	Psize 128
Regularized evolution	0.9396	0.9398	0.9394
PBG (ours)	0.9402	0.9413	0.9410

The regularized evolution algorithm was empirically evaluated to have a mutation rate of 0.72 as optimal. As state-of-the-art baseline models, we found that regularized evolution provides a competitive baseline, which shows robust performance across benchmarks. While newer methods claim state-of-the-art performance on benchmarks such as ^38,39^, we had difficulties reproducing these results, lacking large enough sample sizes in their experiments. A more thorough comparison against newer methods should be part of future work.

### Generalization test on NATS-bench

In order to test the generalization ability of PBG, it was tested on the topology search space of the NATS-Bench. As this benchmark uses a unified encoding of combining both edges and operations, the novel guided mutation approach used the probs0 vector (PBG-0 variant) to sample the mutation index. This allowed us to test the guided mutation in its pure form, avoiding benchmark-specific encoding issues as shown in Section Encoding. We tested our algorithm against the best-performing algorithms of regularized evolution and REINFORCE as implemented in the NATS-Bench paper^34^.

### Ablation studies

In order to differentiate the individual contributions of the two proposed methods of greedy selection and guided mutation, we performed ablation studies. Greedy selection was combined with guided mutation, random mutation that mutates every index with a probability specified by a mutation rate of 0.5, and swapping mutation by interchanging two operations (see Section Greedy selection works best with guided mutation). Guided mutation was combined with greedy selection, random parent pair selection, as well as tournament selection with a tournament size of 10 (see Section Guided mutation benefits from greedy selection). We kept the random-point crossover operation throughout all experiments. For the NAS-Bench-101 specific encoding (see Section Encoding), with loss of generality, we further studied the impact of separately mutating the adjacency matrix and operations by independently sampling mutation indices for each category and combining different approaches of sampling from probs1 or from probs0 (see Section Exploitative guided mutation on operations drives performance gains). We evaluate PBG’s performance for both identical settings (00 and 11) and mixed settings with probs0 for the adjacency matrix and probs1 for operations (01), or vice versa (10). This mixed approach should be considered with caution, as there are benchmark-specific operation biases, as described in Section Encoding, which result in a loss of generality for other benchmarks.

## Results

### Guiding mutation can boost performance even using lower-ranked models as targets

**Fig. 10:**
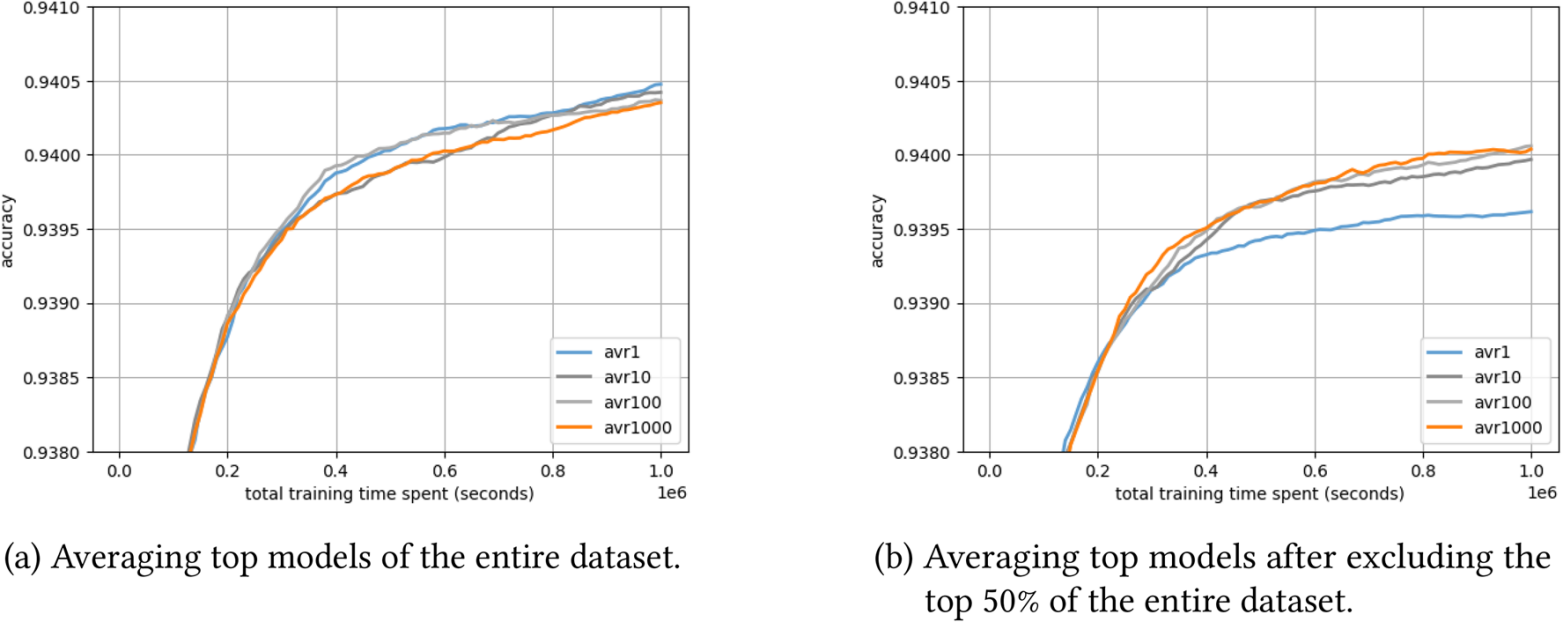
Probability guided mutation model using average distribution of top models from the entire dataset (left) and lower-ranked 50% of the dataset (right).

Figure 10 shows the performance of using the a priori known best models of the NAS-Bench-101 dataset as a guide for the mutation. As expected, Fig. 10a shows better performance when using the best models from the entire dataset as compared to the lower-ranked 50% as in Fig. 10b. This can be explained by the fact that using the top solutions of the entire dataset eventually leads to achieving the best results, having the models eventually mutating to the top solutions.

While using the top-1 model from the full dataset can still provide mutation information, the top-1 model from the remaining dataset cannot boost algorithm performance compared to the other models. Notably, the guided mutation still performs well with the lower-ranked 50% of the dataset, indicating its robustness regardless of the specific models presented. Interestingly, while the top models from the lower-ranked 50% average around 0.906, they can still effectively guide the search toward better solutions in the vicinity of 0.94, almost on par with the top models as targets. Table 2 shows the averaged performance of the top models.

**Table 2:** Averaged performance of top-n models of entire and lower ranked dataset.

	Top 1	Top 10	Top 100	Top 1000
Entire dataset	0.9466	0.9440	0.9418	0.9392
Lower-ranked 50%	0.9055	0.9055	0.9055	0.9055

### Guiding evolution on the current population makes NAS faster and more efficient

Table 3 compares PBG with regularized evolution as a baseline on NAS-Bench-101, showing that PBG-1 outperforms the baseline, reducing the time to reach accuracy milestones by up to 66%.

**Table 3:** Time points (in 1*e*4 s) at which PBG-1, PBG-0, and regularized evolution (RE) reach specified accuracy. Time saved is measured as the reduction of PBG-1 compared to RE.

Accuracy	0.9380	0.9390	0.9400	0.9410	0.9420	0.9425
PBG-1	16	28	48	77	215	432
PBG-0	16	30	55	106	821	–
RE	16	31	86	138	644	1041
Time saved	0%	9%	44%	44%	66%	58%

**Fig. 11:**
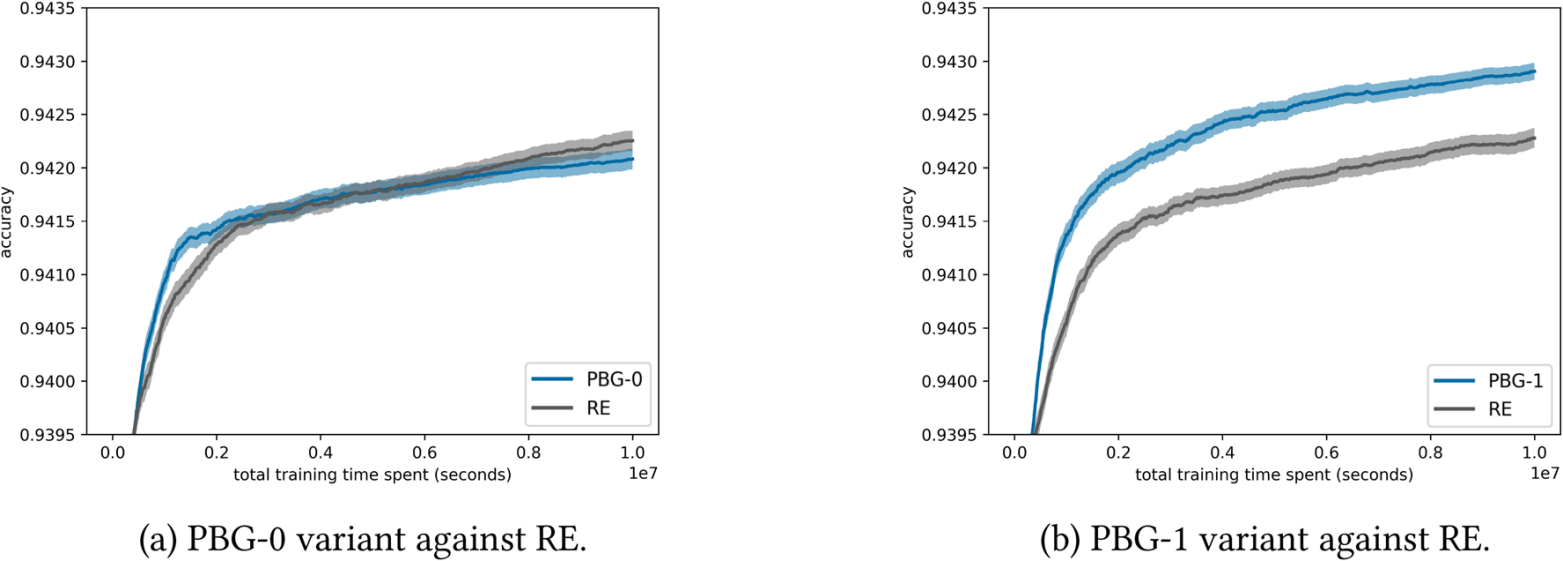
Performance of PBG variants and regularized evolution (RE) on NAS-Bench-101, averaging 1000 runs within a time budget of 1*e*7 seconds with 95% confidence intervals.

A visualization of this is shown in Fig. 11. An advantage of guided mutation is the drastic reduction in hyperparameters that need to be evaluated. While regularized evolution uses a mutation rate and tournament size for selection, PBG relies on a parameter-free solution. On NAS-Bench-101, the exploitative sampling of the probs1 vector in the PBG-1 variant significantly outperforms the baseline, while the PBG-0 variant shows competitive performance compared to the fine-tuned regularized evolution algorithm within the given timeframe. More data on even further extended time budgets can be found in Table 4 in Section Extending time budgets.

### Guiding exploration and exploitation generalizes across benchmarks

**Fig. 12:**
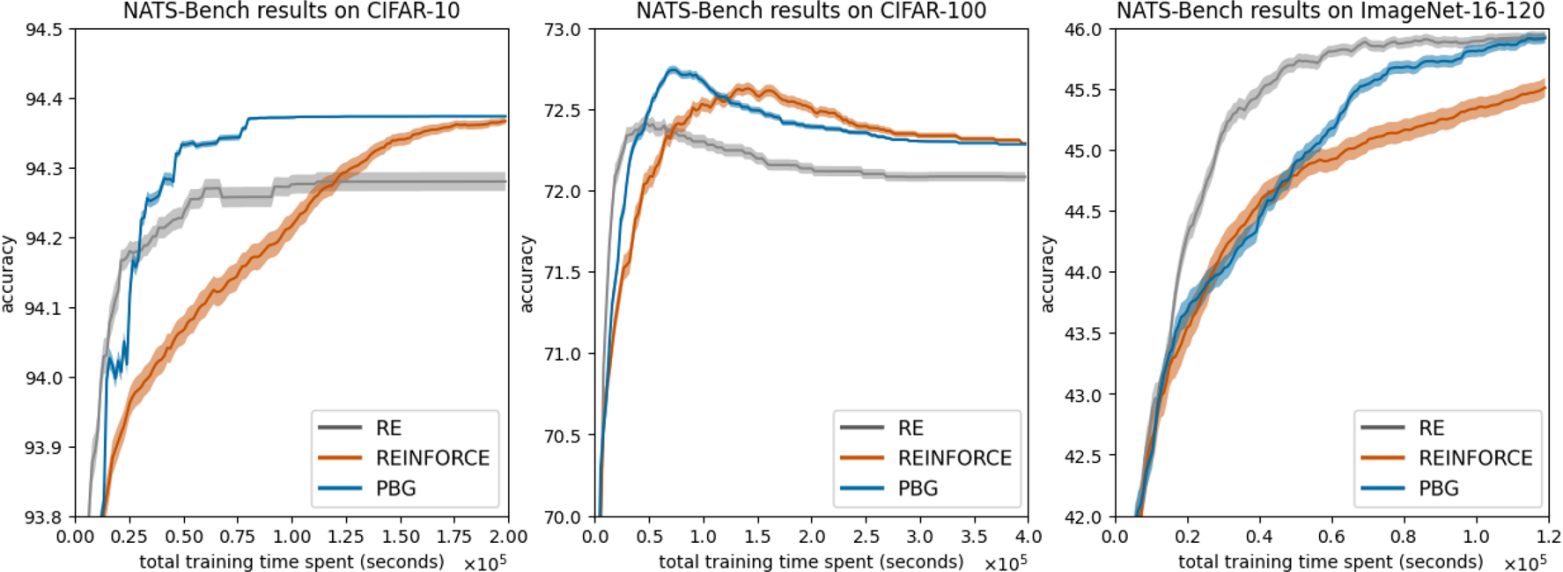
Comparison of PBG-0, RE, and REINFORCE on the NATS-Bench datasets with 95% confidence intervals.

Figure 12 shows the performance of the proposed algorithm in comparison with regularized evolution and REINFORCE on different datasets using the topology search of the NATS-Bench.

The proposed method demonstrates PBG’s generalizability across multiple datasets, namely CIFAR-10, CIFAR-100, and ImageNet16. While it may not consistently show the fastest initial convergence compared to all other methods, it maintains competitive performance (Fig. 12 right), emerges as a top performer (Fig. 12 center), or achieves accelerated convergence within the relevant context (Fig. 12 left).

## Ablation studies

### Greedy selection works best with guided mutation

**Fig. 13:**
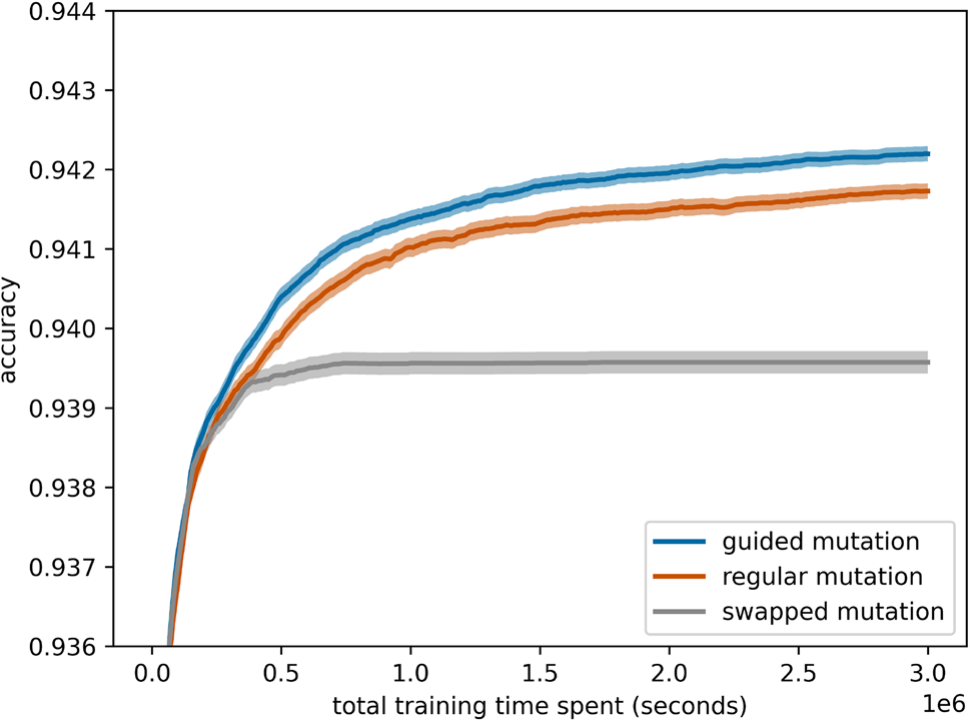
Greedy selection in ablation with regular, swapped, and guided (ours) mutation with 95% confidence intervals.

This ablation study analyzes how different mutation strategies perform when combined with greedy selection. Specifically, Fig. 13 compares the effect of combining greedy selection with regular random mutation (mutating each index with a probability of 0.5), swapping mutation (interchanging two operations), and our proposed guided mutation. The results demonstrate that greedy selection in combination with guided mutation significantly outperforms the baselines. This indicates that while greedy selection focuses on exploitative search by selecting high-performing individuals, guided mutation introduces a complementary exploratory mechanism. This balance between exploration and exploitation leads to superior performance. In contrast, random and swapping mutations lack this targeted exploration, resulting in reduced performance.

### Guided mutation benefits from greedy selection

**Fig. 14:**
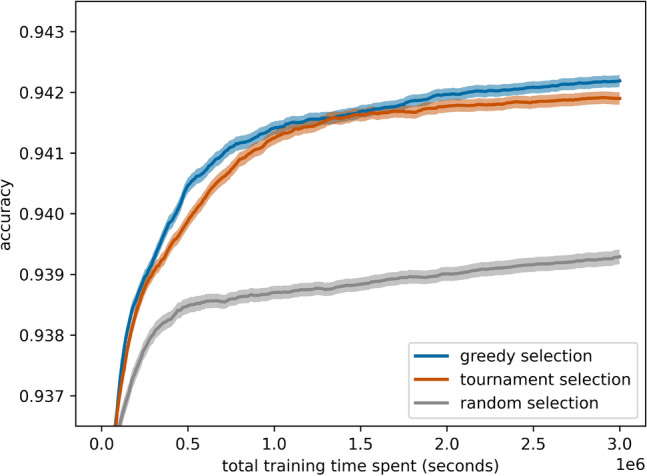
Guided mutation in ablation with random, tournament, and greedy (ours) selection with 95% confidence intervals.

Figure 14 compares the performance of guided mutation under different selection mechanisms: random parent selection, tournament selection (with a tournament size of 10), and greedy selection (ours). The results indicate that guided mutation combined with greedy selection slightly outperforms the combination with tournament selection. Although the difference is marginal, there is a consistent performance boost, suggesting that greedy selection can still enhance the benefits of guided mutation by focusing on high-quality solutions. Nonetheless, the smaller gap implies that guided mutation itself already provides a considerable balance between exploration and exploitation, even when coupled with less selective parent selection strategies.

### Exploitative guided mutation on operations drives performance gains

**Fig. 15:**
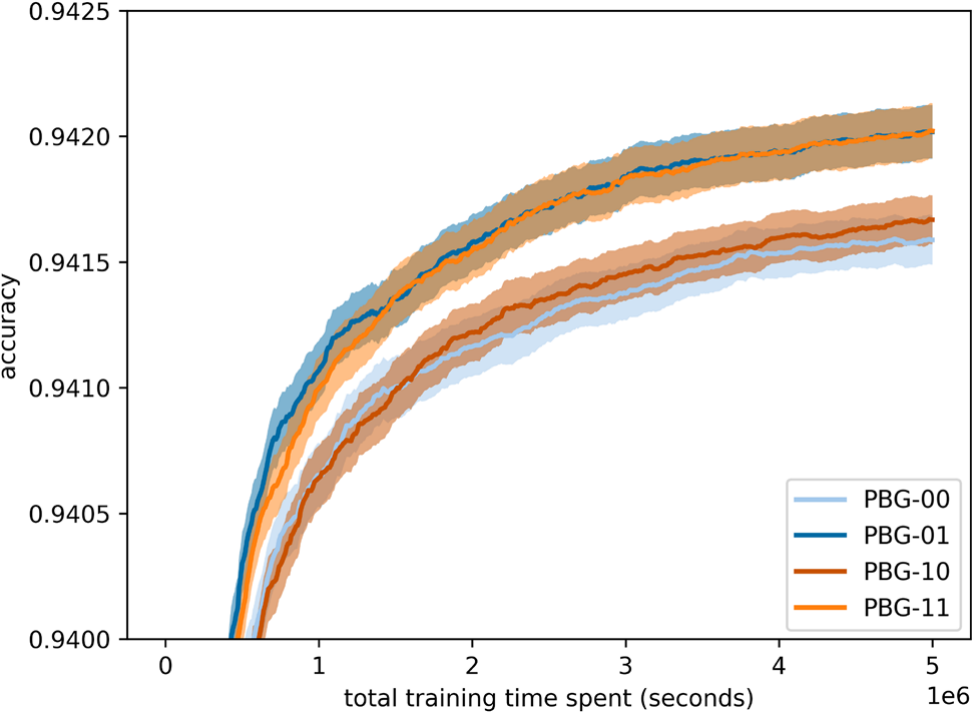
NAS-Bench-101 variants of PBG due to separate adjacency matrix and operation encoding with 95% confidence intervals.

This experiment investigates how separately guiding mutations for the adjacency matrix and operations affect performance on NAS-Bench-101. Figure 15 presents the performance of different combinations of probability vectors used to sample mutations for the adjacency matrix and the operations in NAS-Bench-101-specific encodings.

We evaluate four configurations: 00, 11, 01, and 10, where the first digit corresponds to the adjacency matrix strategy and the second to the operations. The results show that exploitative guided mutation applied to the operations (second digit = 1) is the main driver of performance gains, while the adjacency matrix strategy (first digit) has minimal impact. These findings suggest that exploiting operation probabilities is more beneficial on this benchmark. However, these results should be taken with caution, as the benchmark-specific operation biases discussed in Section Encoding may have influenced the outcome, limiting generalizability to other benchmarks.

## Extending time budgets

Adhering to best practices of extending time budgets until convergence^36^, we extended the originally used time budget of 1*e*7 to 1*e*8 seconds for the NAS-Bench-101. In an even larger time frame of 1*e*8 seconds, the regularized evolution still shows superior performance after about 2*e*7 seconds, which is equivalent to 231 GPU days of simulation time.

**Fig. 16:**
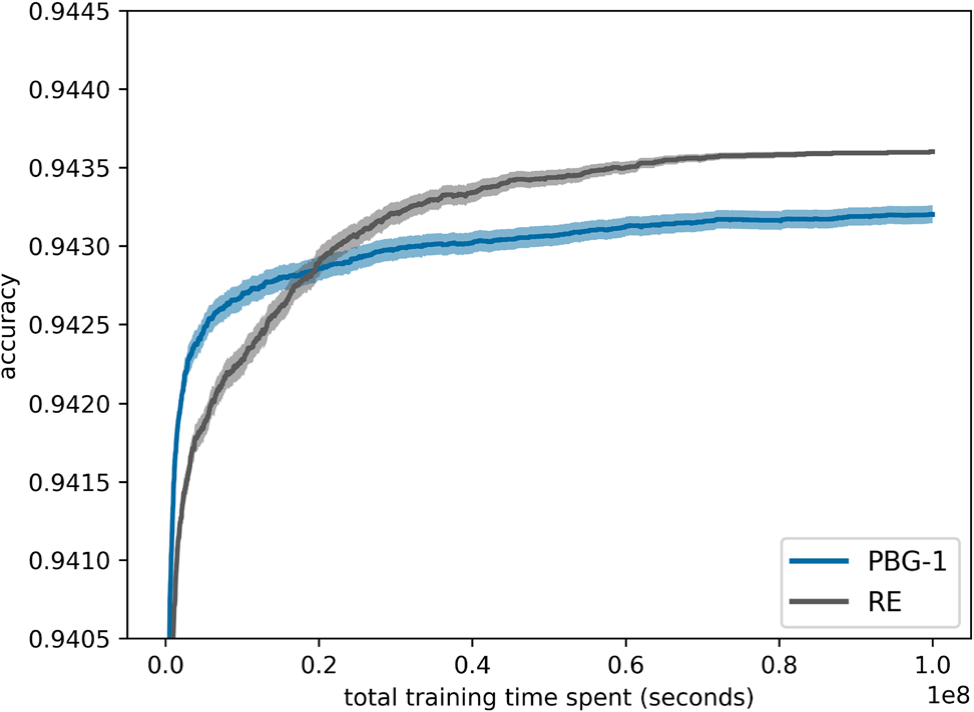
PBG-1 and regularized evolution (RE) on NAS-Bench-101 within 1*e*8 seconds with 95% confidence intervals.

**Table 4:** Mean accuracy (std in 1*e*-2) averaging 1000 runs of PBG-1 and regularized evolution (RE).

Time (s)	1*e*5	1*e*6	1*e*7	2*e*7	1*e*8
PBG-1	0.9370 (0.26)	0.9414 (0.18)	0.9427 (0.14)	0.9429 (0.12)	0.9432 (0.09)
RE	0.9370 (0.27)	0.9405 (0.20)	0.9423 (0.16)	0.9429 (0.14)	0.9436 (0.02)

## Discussion

In this study, we have shown that sampling mutation indices from the population distribution of a given generation as a probability vector proved effective in sampling new generations. A novel guided mutation algorithm, which samples from the population distribution, outperformed the presented competitive baselines. Population-Based Guiding (PBG) integrates an exploiting greedy selection and explorative guided mutation. Outperforming the regularized evolution method on NAS-Bench-101, PBG solves the gap by enhancing the efficiency in NAS. Testing PBG on the NATS-Bench further demonstrated its generalizability and competitive performance across multiple datasets, including CIFAR-10, CIFAR-100, and ImageNet16. These findings demonstrate the potential of PBG to significantly enhance NAS efficiency and effectiveness, laying a robust foundation for future research and development in this domain.

The effectiveness of PBG arises from its ability to adaptively balance exploration and exploitation without introducing additional hyperparameters or manual heuristics. Traditional evolutionary NAS methods rely on fixed mutation probabilities or random perturbations, which often result in inefficient or redundant sampling of the search space. In contrast, PBG was deliberately designed to transform population-level information into a self-guiding mechanism: by sampling mutation indices from the population’s fitness distribution, the algorithm implicitly learns which regions of the search space are promising while still maintaining stochasticity for diversity. This design eliminates human bias at the meta-level–the definition of mutation and selection strategies–and enables a more autonomous and data-driven evolutionary process.

A common concern is that a greedy selection strategy can easily lead to suboptimal solutions, as it favors immediate exploitation over long-term exploration, which might appear counterintuitive for an evolutionary search. This may help explain that regularized evolution eventually outperforms PBG under longer time budgets, despite initial appearances that a greedy approach should perform well. However, in PBG this potential drawback is intentionally counterbalanced by the guided mutation mechanism. While greedy selection accelerates convergence by consistently recombining the fittest individuals, guided mutation introduces stochastic exploration based on intra-generational population statistics. This ensures that while the algorithm prioritizes high-performing parent combinations, it simultaneously injects novelty from underexplored areas of the search space. Consequently, what appears to be a short-sighted greedy approach becomes an effective synergistic driver of convergence and diversity, allowing the search to progress efficiently without stagnating in local optima.

The parameter-free nature of the guided mutation was designed to improve reproducibility and reduce the tuning overhead that commonly hampers NAS research. By grounding mutation behavior in the current population rather than external hyperparameters, PBG dynamically adapts its search strategy over time, yielding both efficiency and generalization across benchmarks. Ultimately, PBG’s design philosophy is rooted in the principle of minimizing human bias while maximizing algorithmic adaptability. Instead of encoding fixed rules for exploration and exploitation, it leverages emergent properties of population dynamics to learn how to evolve more efficiently. This explains not only why PBG performs effectively but also why its seemingly counterintuitive combination of greedy exploitation and guided exploration leads to a self-regulating, data-informed search paradigm capable of robust performance without the need for hyperparameters.

However, we need to acknowledge some limitations of our work. The NAS-Bench-101 is limited to a separate encoding for adjacency matrix and operations, which can result in a biased exploration of suitable operations (Fig. 5). The shadowed nodes introduce a benchmark-specific anomaly that can cause effects on different mutation algorithms when handling shadowed operations (See Section Shadowed nodes). Resolving these issues with a unified approach could facilitate more straightforward algorithm comparisons. Future efforts should aim to develop benchmarks with consistent encodings. Adopting a generic architecture representation, ideally in a fixed embedded dimension space, could streamline algorithm comparisons across different benchmarks.

When benchmarking PBG, the maximum time frame for conducting NAS runs continues to yield inconsistent results. While PBG notably outperforms regularized evolution within an extended time frame compared to the original paper’s test, further extension of this timeframe reverses the trend, with regularized evolution outperforming PBG eventually (Fig. 16). A comprehensive exploration of mixed and true one-hot encoding approaches may shed new light on the exploration-exploitation tradeoff by sampling from both the population distribution and its inverse. Although the true one-hot approach selects only one index per mutation operation, and the mixed approach utilizes two indices for matrix and operation selection, the introduction of a hyperparameter could enhance performance by regulating the number of mutations.

While we achieve, to the best of our knowledge, competitive and robust performance within evolutionary NAS, other methods that do not involve evolutionary strategies achieve promising and superior results while requiring less computational power.

Although recent advancements in NAS algorithms have shown significant potential for improvement^13,38^, future work should prioritize developing methods to effectively train the mutation process. For instance, a Transformer-based model^40^ could be designed to propose new individuals by weighing all previous candidates, incorporating a highly generic representation of individuals as proposed in ^41^. This approach would enable the model to transfer knowledge from prior runs, thus accelerating the search process on unseen datasets similar to a meta-learned genetic algorithm^24^.

## Data Availability

The code and data used in the study are available at https://github.com/ankilab/PBG.
